# The Effect of Pre-Analytical Variability on the Measurement of MRM-MS-Based Mid- to High-Abundance Plasma Protein Biomarkers and a Panel of Cytokines

**DOI:** 10.1371/journal.pone.0038290

**Published:** 2012-06-06

**Authors:** Adriana Aguilar-Mahecha, Michael A. Kuzyk, Dominik Domanski, Christoph H. Borchers, Mark Basik

**Affiliations:** 1 Department of Oncology, Lady Davis Institute, McGill University, Montreal, Quebec, Canada; 2 Genome BC Proteomics Centre, University of Victoria, Victoria, British Columbia, Canada; 3 Department of Biochemistry and Microbiology, University of Victoria, Victoria, British Columbia, Canada; Ludwig-Maximilians-University Munich, Germany

## Abstract

Blood sample processing and handling can have a significant impact on the stability and levels of proteins measured in biomarker studies. Such pre-analytical variability needs to be well understood in the context of the different proteomics platforms available for biomarker discovery and validation. In the present study we evaluated different types of blood collection tubes including the BD P100 tube containing protease inhibitors as well as CTAD tubes, which prevent platelet activation. We studied the effect of different processing protocols as well as delays in tube processing on the levels of 55 mid and high abundance plasma proteins using novel multiple-reaction monitoring-mass spectrometry (MRM-MS) assays as well as 27 low abundance cytokines using a commercially available multiplexed bead-based immunoassay. The use of P100 tubes containing protease inhibitors only conferred proteolytic protection for 4 cytokines and only one MRM-MS-measured peptide. Mid and high abundance proteins measured by MRM are highly stable in plasma left unprocessed for up to six hours although platelet activation can also impact the levels of these proteins. The levels of cytokines were elevated when tubes were centrifuged at cold temperature, while low levels were detected when samples were collected in CTAD tubes. Delays in centrifugation also had an impact on the levels of cytokines measured depending on the type of collection tube used. Our findings can help in the development of guidelines for blood collection and processing for proteomic biomarker studies.

## Introduction

The detection and quantification of circulating proteins in the blood of patients has great potential for the development of clinically useful biomarkers. Blood has always been considered an attractive source of biomarkers because it comes in contact with all of the tissues in the body, and factors can be released from these tissues into the circulation. Moreover, because blood is easily accessible and its collection is inexpensive and minimally invasive, it is ideal for clinical assays. Plasma and serum protein biomarkers are already used for diagnosis, prediction, and monitoring of response to treatment in many diseases, including cancer [1]. The discovery of blood-based protein biomarkers is very challenging due to the complexity of the plasma and serum proteome, and because there is a difference of 10 orders of magnitude between albumin and the least abundant plasma protein, which poses a major hurdle to the discovery and validation of low-abundance biomarkers [2]. Lately, with the development of more sensitive, accurate and high-throughput proteomics technologies, there has been a substantial increase in the number of candidate protein biomarkers reported in plasma and serum from cancer patients [3]. For instance, the use of multiple-reaction monitoring-mass spectrometry (MRM-MS) has provided a novel and very precise method for measuring protein biomarkers in complex tissues such as blood, obviating the requirement for immunodepletion, although it is still limited in sensitivity to proteins present in nanogram/ml concentrations.

The success of any biomarker study will depend in large part on the quality of the biospecimen analyzed, and on the control of factors that may introduce bias to the study even before the sample reaches the analytical platform. In the present study, we focus on the issue of pre-analytical variability, which can be introduced at various steps while the sample is being collected and processed, and which has been identified as a major source of bias in proteomics studies [4]. Some of the factors that are potential sources of pre-analytical variation in blood clinical proteomic studies include: the type of blood collection tube used (serum, plasma, and use of additives), sample handling and processing, time elapsed between sample collection and processing and between sample processing and storage, and repetitive freeze/thaw cycles of the samples [5], [6].

Due to all this potential variability, standardization of protocols for blood collection and processing is essential, especially when sample collection takes place at multiple clinical sites [7], [8]. However, a universal set of recommendations is very unlikely to be proposed until the nature and magnitude of the variability inherent in serum and plasma proteomics studies is fully understood.

In order to address some of the sources of this variability (e.g. the activation of blood proteases and platelet degranulation), manufacturers have produced novel blood collection tubes. Our study is an independent assessment of the impact of these tubes on pre-analytical variability by measuring defined plasma proteins. We analyze blood collected in tubes containing protease inhibitors, BD™ P100 Blood Collection Tubes, which have been developed to stabilize proteins and minimize proteolytic degradation during the collection and processing of blood specimens. We also analyze blood collected in CTAD tubes, which prevent platelet degranulation/activation so as to minimize the exogenous release of proteins from platelets, which could have a significant impact on the level of circulating plasma proteins. We assess how different processing protocols and delays from the time of collection to sample processing can affect the levels of low-abundance cytokines as well as mid- to high-abundance plasma proteins. We performed our studies using a commercially-available multiplexed bead-based immunoassay(Bio-Plex® Suspension Array System), and, for the first time, using a 55-protein panel on a MRM-MS platform.

## Methods

### Study Samples

Blood was collected from 14 fasting healthy individuals (7 females and 7 males, age range 22 to 50 years) who provided written informed consent to participate in this study. The local Ethics Board of the Jewish General Hospital (Montreal, Canada) approved this study. Blood from each seated volunteer was collected by a trained nurse. The blood was collected directly into different blood collection tubes by a single venipuncture using the BD Vacutainer® Safety-Lok™ Blood Collection Set (BD part # 367283). We tested three different types of blood collection tubes: 4 ml k_2_EDTA tubes (BD part # 366450), 8 ml k_2_EDTA tubes containing protease inhibitors (BD™ P100 Blood Collection Tubes, BD part #366456) as well as 4.5 ml CTAD tubes (BD part # 367947) which contain citrate as the anticoagulant and a mixture of theophylline, adenosine, and the light-sensitive agent dipyrimadole, which all contribute to reduce platelet activation [9]. Each tube was filled with the required volume of blood and gently inverted ten times to allow for appropriate mixing of anticoagulants and tube additives. CTAD tubes were wrapped in aluminum foil until centrifugation to protect from light. For the time course studies, samples were immediately centrifuged (T0), or left on the bench for 2 hrs (T2) or 6 hrs (T6) and processed immediately thereafter. Samples were processed according to different protocols designated A, B, C and D (Table 1). Protocol A: samples were processed at 2000×*g* for 5 minutes in a refrigerated (8°C) swing-type centrifuge (initial guidelines recommended by the manufacturer for centrifuging P100 tubes). Protocol B: samples were swing centrifuged for 20 minutes at 2500×*g* at room temperature (Updated guidelines recommended by the manufacturer for centrifuging P100 tubes). Protocol C: samples were processed in a swing-type centrifuge at 1300×*g* for 10 minutes; plasma was collected and centrifuged again in a countertop microcentrifuge at 2500×*g* for 15 minutes to remove any potentially-remaining cellular material. Both centrifugations were done at room temperature. Protocol D: samples were centrifuged at 1300×*g* for 10 minutes at room temperature; plasma was collected and passed through a 0.45 µm nylon filter to remove any potentially-remaining cellular material.

**Table 1:** Blood tubes and processing protocols.

Tube	Protocol	RCF (x *g*)	Time (minutes)	Temperature
P100	A	2000	5	8°C
P100	B	2500	20	RT
P100	C	1300†	10	RT
		2500‡	15	RT
kEDTA	C	1300†	10	RT
		2500‡	15	RT
CTAD	D	1300	10	RT

† First centrifugation.

‡ Second centrifugation.

All samples collected were aliquoted in cryovials (250 µl/−aliquot) and stored at −80°C immediately after processing. The maximum time from the time of collection to the time of storage at −80°C was 1 hour. Samples were never thawed until immediately before analysis to avoid any effect of freeze/thawing.

### MRM Analysis

A 3-µL aliquot of each plasma sample was used to prepare plasma tryptic digests. Samples were diluted and denatured by the addition of 174.5 µL of 25 mM ammonium bicarbonate (AMB) and 30 µL of 10% w/v sodium deoxycholate (NaDOC) [10]. Denatured plasma samples were reduced with 5 mM tris(2-carboxyethyl)phosphine (TCEP) and incubated at 60°C for 30 min. Free sulfhydryl groups were alkylated with 10 mM iodoacetamide for 30 min at 37°C in the dark. Next, 10.5 µL of modified porcine trypsin solution (0.4 µg/µL in 25 mM AMB, Promega Gold Sequencing Grade) was added to each sample to give a final digest volume of 300 µL. Samples were digested for 16 h at 37°C. Digestion was stopped by the addition of acidified SIS peptides (Top45 SIS peptide cocktail [11] except the peptide encoding for transferrin, plus 11 additional peptides including two new peptides encoding for transferrin (Table S1)) in formic acid to produce a final formic acid concentration of 0.5% v/v. Samples were centrifuged for 5 min at 13,000×g (23°C) to remove NaDOC precipitate, and the supernatant was desalted and concentrated by solid phase extraction using Waters Oasis HLB Elution plates (2 mg). The eluted samples were vacuum concentrated to dryness and rehydrated in a volume of Solvent A (0.1% v/v formic acid) corresponding to a 1/70 dilution of plasma prior to LC-MS/MS analysis. LC-MRM/MS analysis of plasma digests was performed as previously reported [11]. Briefly, the LC-MRM-MS system consisted of an Eksigent NanoLC-1Dplus interfaced to an Applied Biosystems (AB)/MDS Sciex 4000 QTRAP. The MS was equipped with a nanoelectrospray ionization source and was controlled by AB’s Analyst 1.5 software. A reversed-phase capillary column (75 µm×15 cm) packed in house using Magic C18AQ (5-µm diameter particles, 100-Å pore size; Michrom, Auburn, CA), was used for the on-line separations, at a flow rate of 300 nl/min. The gradient used consisted of a six minute hold at 100% solvent A (2% acetonitrile, 0.1% formic acid), followed by a 32-min linear gradient to 23% B, and then a 9-min linear gradient to 43%B. The MS acquisition parameters were: positive ion mode; ion spray voltage,1900–2000 V; curtain gas, 25 p.s.i.; interface temperature, 200°C; collision-induced dissociation (CID) pressure, 3.5×10^5^ torr; and Q1 and Q3 set to unit resolution (0.6–0.8-Da full width at half-height). MRM acquisition methods (60 min in length) were constructed using 3 MRM ion pairs per peptide (336 MRM ion pairs total) using peptide-specific tuned Declustering Potential and Collision Energy voltages and retention time constraints (Table S2). A default collision cell exit potential of 23 V was used for all MRM ion pairs, and the scheduled MRM option was used for all data acquisition with a target cycle time of 1 second and a 5 min MRM detection window. Blank solvent injections (30 min analyses) were analyzed between all patient samples to prevent sample carryover on the HPLC column.

### MRM Data Analysis

We used a concentration balanced SIS-peptide mixture to normalize peak areas against the peak areas of co-eluting SIS peptides [11]. All MRM data was processed using MultiQuant™ Software Version 1.1 (Applied Biosystems) with the MQL algorithm for peak integration. A 2-min retention-time window, with “report largest peak” enabled and a 1-point Savitsky-Golay smooth with a peak-splitting factor of 2, was used. The default MultiQuant™ values for noise percentage and baseline subtraction window were used. All data was manually inspected to ensure correct peak detection and accurate integration. Linear regression of all calibration curves was performed using a standard 1/× (x = concentration ratio) weighting option to aid in covering a wide dynamic range.

### Cytokine Bio-Plex Assay

We used the Bio-Plex® Suspension Array System (Bio-Rad, CA), a bead based multiplex immunoassay [12], and the 27-plex human cytokine panel (Bio-Rad part # M50-0KCAF0Y) to measure the levels of IL-1β, IL-1 receptor antagonist (IL-1RA), IL-2, IL-4, IL-5, IL-6, IL-7, IL-8, IL-9, IL-10, IL-12 (p70), IL-13, IL- 15, IL-17, Eotaxin, FGF-b, granulocyte colony-stimulating factor (G-CSF), GM-CSF, interferon (IFN)-γ, IP-10, monocyte chemoattractant protein (MCP)-1, macrophage inflammatory protein (MIP)-1α, MIP-1β, platelet-derived growth factor BB (PDGF-BB),regulated on activation normal T-cell expressed and secreted (RANTES), tumour necrosis factor (TNF), and vascular endothelial growth factor (VEGF). Whole plasma was diluted 4× in human serum diluent, and the assay was performed according to the manufacturer’s guidelines. Samples were measured in duplicate and read with the Luminex® 200™ system (Bio-Rad Laboratories, Hercules, CA). Data was analysed using Bio-Plex Manager™ Software Version 4.0 (Bio-Rad, CA). The calibration curve for each cytokine was analysed with both 4PL and 5PL logistic regression curves and the best fit curve was chosen for further analyses. Data was considered reliable if at least 5 data points from the calibration curve fell within 70–130% of the expected values as recommended by the manufacturer. Two separate Bio-Plex® runs were performed. By applying the above criteria, we could reliably measure 21 cytokines and 20 cytokines in the first and second Bio-Plex® experiments, respectively.

### Statistical Analyses

For statistical purposes, cytokines that were below the level of detection of the Bio-Plex® assay were assigned an arbitrary value corresponding to half of the limit of detection (LOD) of the analyte. We used two-tailed paired *t* tests to make comparisons between tube types and between time points. One-Way ANOVA repeated measures and Tukeys Post-hoc method [13] was used for the time-course analyses. All statistical analyses were done with GraphPad Prism Version 5 (San Diego, CA).

## Results

### Effect of Collection Tube and Delays in Processing of Blood Sample on Mid– to High Abundance Plasma Proteins as Determined by MRM

To study the effect of blood collection tube and delays in processing on the measurement of medium to high abundance plasma proteins, we used multiple reaction monitoring (MRM) and stable isotope-labeled standard peptides. With this approach, we were able to measure plasma levels of 55 mid- to high-abundance plasma proteins. For this assay, we analysed plasma collected in P100 tubes, k_2_EDTA tubes and CTAD tubes all processed with comparable protocols (C and D) (Table 1). We also performed a time-course study and samples were processed at time 0, 2 hours or 6 hours following collection. The reproducibility of each individual MRM assay was assessed and the %CV for all the peptides was below 20% with 51 peptides having %CVs below 10% (Table S1).

With MRM-MS, we could not identify any differences in the levels of tryptic peptides measured in plasma collected in k_2_EDTA tubes with (P100 tubes) or without protease inhibitors at any of the time points analysed. In fact, only one peptide, proteotypic for plasminogen, showed statistically higher levels at the 6 h time point in k_2_EDTA relative to P100, but the percent difference in concentration was minimal (only 4%) (Table 2). In contrast, blood collection in CTAD tubes resulted in significant differences in the levels of peptides measured when compared to the other collection tubes used. The most common finding was significantly lower (p<0.05) levels of peptides in samples collected in CTAD tubes. Of the 56 peptides analysed, lower levels were observed for 21 peptides in samples processed at time 0, 28 peptides in samples processed at time 2 h, and 41 peptides in samples processed at time 6 h; in CTAD tubes compared to all other tube types (Table 3). Of the 21 peptides with lower levels at time 0 in CTAD tubes, the levels of 7 were lower relative to both P100 and k_2_EDTA tubes; the levels of 8 were lower relative to k_2_EDTA tubes only and the levels of 6 were lower relative to P100 tubes only (the percent decreases ranged from 10%–48%). Of the 28 peptides with lower levels in CTAD samples processed at time 2 hours, 7 presented lower levels in CTAD samples relative to both P100 and k_2_EDTA tubes and the levels of 21 peptides were lower in CTAD tubes relative to P100 tubes only (the percent decreases ranged from 11%–45%). Finally, of the 41 peptides with lower levels in CTAD samples processed at time 6 h, 16 had lower levels relative to both P100 and k_2_EDTA samples, 4 were lower in CTAD relative to k_2_EDTA only and 21 were lower in CTAD relative to P100 tubes only (the percent decreases ranged from 9%–47%) (Table 3).

**Table 2:** Percent differences in peptide levels between tube types measured by MRM.

		Time 0	Time 2	Time 6
		P100:k-EDTA	P100:k-EDTA	P100:k-EDTA
Protein	Peptide	%	%	%
Adiponectin	IFYNQQNHYDGSTGK	12.28	−13.79	5.25
Afamin	DADPDTFFAK	10.70	−2.47	−4.38
Albumin, serum	LVNEVTEFAK	4.35	−1.29	−0.81
Alpha-1-acid glycoprotein 1	NWGLSVYADKPETTK	20.15	11.24	9.91
Alpha-1-antichymotrypsin	EIGELYLPK	2.33	0.42	−0.26
Alpha-1-Anti-trypsin	ITPNLAEFAFSLYR	−4.00	−10.49	−7.75
Alpha-1B-glycoprotein	LETPDFQLFK	2.62	−6.00	−2.99
Alpha-2-antiplasmin	LGNQEPGGQTALK	9.98	−1.59	5.18
alpha-2-HS-glycoprotein	HTLNQIDEVK	2.80	−0.60	1.80
alpha-2-macroglobulin	TEHPFTVEEFVLPK	6.51	−2.50	−3.71
Angiotensinogen	ALQDQLVLVAAK	6.46	−5.29	0.71
Antithrombin-III	DDLYVSDAFHK	6.59	−1.67	10.56
Apolipoprotein A-I	ATEHLSTLSEK	6.53	−2.44	−1.68
Apolipoprotein A-II precursor	SPELQAEAK	9.27	2.98	3.33
Apolipoprotein A-IV	SLAPYAQDTQEK	9.23	1.02	−3.01
Apolipoprotein B-100	FPEVDVLTK	1.26	−3.03	−2.50
Apolipoprotein C-I lipoprotein	TPDVSSALDK	10.39	0.70	−3.01
Apolipoprotein C-III	GWVTDGFSSLK	19.42	9.11	5.62
Apolipoprotein D	IPTTFENGR	−5.55	−4.96	−6.50
Apolipoprotein E	LGPLVEQGR	5.73	4.36	−4.90
Apolipoprotein L1	VTEPISAESGEQVER	6.26	−3.35	−20.92
Beta-2-glycoprotein I	ATVVYQGER	3.03	−2.52	−2.94
Ceruloplasmin	EYTDASFTNR	6.80	−2.10	−10.93
Clusterin	ELDESLQVAER	7.74	−0.91	−11.37
Coagulation factor XIIa HC	VVGGLVALR	7.77	−8.25	−0.50
Coagulation Factor XIII (a chain)	STVLTIPEIIIK	−6.10	10.46	23.94
Complement C1 inactivator	LLDSLPSDTR	6.04	−1.15	−0.60
Complement C3	TGLQEVEVK	5.50	−3.33	0.41
Complement C4 beta chain	VDGTLNLNLR	4.03	−2.61	−6.41
Complement C4 gamma chain	ITQVLHFTK	6.53	−3.41	−3.63
Complement C9	LSPIYNLVPVK	6.81	−1.30	−3.11
Complement factor B	EELLPAQDIK	7.34	−0.36	−5.34
Complement factor H	SPDVINGSPISQK	10.09	5.93	2.86
Fibrinogen alpha chain	GSESGIFTNTK	9.46	1.18	0.34
Fibrinogen beta chain	QGFGNVATNTDGK	11.35	10.59	−1.90
Fibrinogen gamma chain	YEASILTHDSSIR	13.78	−4.11	3.84
Fibrinopeptide A	ADSGEGDFLAEGGGVR	10.86	3.36	3.37
Fibronectin	VPGTSTSATLTGLTR	28.53	−7.64	−11.19
Gelsolin, isoform 1	TGAQELLR	7.00	−5.23	−3.76
Haptoglobin beta chain	VGYVSGWGR	2.42	−2.64	−3.40
Hemopexin	NFPSPVDAAFR	4.70	−2.32	−2.09
Heparin cofactor II	TLEAQLTPR	−1.88	−0.14	−5.56
Histidine-rich glycoprotein	DGYLFQLLR	8.97	−1.61	−6.50
Inter-alpha-trypsin inhibitor HC	AAISGENAGLVR	11.27	−6.15	−2.31
Kininogen-1	TVGSDTFYSFK	5.66	−2.72	−2.81
L-selectin	AEIEYLEK	10.00	3.72	1.55
Plasma retinol-binding protein	YWGVASFLQK	5.17	−3.22	3.19
Plasminogen	LFLEPTR	10.24	0.80	**4.44** *
Prothrombin	ETAASLLQAGYK	13.42	−0.43	8.03
Serum amyloid P-component	VGEYSLYIGR	19.48	11.13	3.98
Transferrin	EGYYGYTGAFR	4.68	−46.05	−4.18
	HSTIFENLANK	6.33	70.23	−1.33
Transthyretin	AADDTWEPFASGK	17.32	1.05	3.47
Vitamin D-binding protein	THLPEVFLSK	8.07	−9.36	4.24
Vitronectin	FEDGVLDPDYPR	4.53	−5.85	−1.99
Zinc-alpha-2-glycoprotein	EIPAWVPFDPAAQITK	−2.67	1.53	−5.03

* p<0.05.

** p<0.001.

**Table 3:** Percent differences in peptides measured in P100 and kEDTA tubes compared to CTAD tubes.

		Time 0	Time 2	Time 6	Time 0	Time 2	Time 6
		P100:CTAD	P100:CTAD	P100:CTAD	k-EDTA:CTAD	k-EDTA:CTAD	k-EDTA:CTAD
Protein	Peptide	%	%	%	%	%	%
Adiponectin	IFYNQQNHYDGSTGK	−**33.66** *	−**41.68** *	−37.65	−**40.92** *	−**32.35** *	−**40.77** *
Afamin	DADPDTFFAK	−5.17	−**13.39** *	−**13.78** *	−14.33	−11.20	−**9.83** *
Albumin, serum	LVNEVTEFAK	−9.37	−**15.15** *	−**17.62** *	−13.14	−14.04	−**16.95** **
Alpha-1-acid glycoprotein 1	NWGLSVYADKPETTK	**60.99** *	42.55	**46.02** *	33.99	28.14	32.86
Alpha-1-antichymotrypsin	EIGELYLPK	−9.69	−13.96	−13.36	**−11.74** *	−14.32	**−13.14** *
Alpha-1-Anti-trypsin	ITPNLAEFAFSLYR	**−48.11** **	**−45.23** **	**−46.95** **	**−45.96** **	**−38.81** **	**−42.49** **
Alpha-1B-glycoprotein	LETPDFQLFK	−11.53	−**15.86** *	−**15.82** *	−13.78	−10.49	−13.23
Alpha-2-antiplasmin	LGNQEPGGQTALK	−6.79	−5.87	−**12.09** *	−15.25	−4.34	−**16.43** *
alpha-2-HS-glycoprotein	HTLNQIDEVK	−9.61	−13.69	−14.43	−12.07	−13.16	−15.94
alpha-2-macroglobulin	TEHPFTVEEFVLPK	−6.94	−15.10	−**16.08** *	−12.63	−12.92	−12.84
Angiotensinogen	ALQDQLVLVAAK	−4.69	−**22.73** *	−**17.35** *	−10.48	−18.42	−17.94
Antithrombin-III	DDLYVSDAFHK	**22.47** *	8.27	**17.34** *	**14.90** *	10.11	6.13
Apolipoprotein A-I	ATEHLSTLSEK	−9.11	−**16.52** *	−**16.87** *	−14.68	−14.43	−15.45
Apolipoprotein A-II precursor	SPELQAEAK	**12.16** *	7.45	**5.61** *	2.64	4.34	2.21
Apolipoprotein A-IV	SLAPYAQDTQEK	−**10.38** *	−**16.64** *	−**21.48** **	−17.95	−17.48	−19.04
Apolipoprotein B-100	FPEVDVLTK	−13.50	−**13.31** *	−**16.38** *	−**14.57** *	−10.60	−**14.23** *
Apolipoprotein C-I lipoprotein	TPDVSSALDK	−7.61	−**13.77** *	−**18.59** **	−16.30	−14.37	−16.07
Apolipoprotein C-III	GWVTDGFSSLK	**19.18** *	11.87	**9.70** *	−0.20	2.53	3.87
Apolipoprotein D	IPTTFENGR	**−21.98** *	**−13.35** *	**−23.83** *	**−**17.40	**−**8.83	**−**18.54
Apolipoprotein E	LGPLVEQGR	**−14.13** *	**−13.34** *	**−26.27** **	**−**18.78	**−**16.96	**−**22.47
Apolipoprotein L1	VTEPISAESGEQVER	**−**19.13	10.28	**−**38.92	**−**23.90	14.10	**−**22.76
Beta-2-glycoprotein I	ATVVYQGER	**−**7.84	**−16.55** *	**−14.69** *	**−**10.55	**−**14.39	**−**12.11
Ceruloplasmin	EYTDASFTNR	0.89	**−**1.93	**−15.68** *	**−**5.53	0.17	**−**5.33
Clusterin	ELDESLQVAER	**−19.87** *	5.43	**−**38.56	**−25.62** *	6.40	**−30.68** *
Coagulation factor XIIa HC	VVGGLVALR	**−**6.27	**−14.88** *	**−15.31** *	**−**13.02	**−**7.22	**−**14.89
Coagulation Factor XIII (a chain)	STVLTIPEIIIK	**−**11.04	**−**2.93	**−**2.46	**−**5.26	**−**12.12	**−**21.30
Complement C1 inactivator	LLDSLPSDTR	**−20.12** *	**−28.38** *	**−25.15** *	**−24.67** *	**−27.55** *	**−24.69** *
Complement C3	TGLQEVEVK	**−11.69** *	**−18.59** *	**−19.78** *	**−16.30** *	**−15.79** *	**−20.10** *
Complement C4 beta chain	VDGTLNLNLR	**−21.82** *	**−22.86** *	**−30.48** *	**−**24.85	**−**20.79	**−**25.72
Complement C4 gamma chain	ITQVLHFTK	**−21.63** *	**−30.71** *	**−28.59** *	**−**26.44	**−**28.27	**−**25.90
Complement C9	LSPIYNLVPVK	**−**10.37	**−14.24** *	**−16.64** *	**−16.08** *	**−**13.11	**−13.96** *
Complement factor B	EELLPAQDIK	**−**0.98	**−**10.32	**−**8.84	**−**7.75	**−**9.99	**−**3.70
Complement factor H	SPDVINGSPISQK	**−**6.69	**−**16.75	**−**13.62	**−**15.24	**−**21.41	**−16.01** *
Fibrinogen alpha chain	GSESGIFTNTK	**−**10.63	**−16.41** *	**−22.91** *	**−18.35** *	**−17.39** *	**−23.17** *
Fibrinogen beta chain	QGFGNVATNTDGK	**−13.17** *	−3.50	**−27.57** *	−22.02	−12.74	**−26.17** *
Fibrinogen gamma chain	YEASILTHDSSIR	−8.72	**−15.29** *	**−17.34** *	−19.77	**−11.66** *	**−20.4** *
Fibrinopeptide A	ADSGEGDFLAEGGGVR	−11.55	−12.23	**−18.99** *	**−20.22** *	−15.09	**−21.63** *
Fibronectin	VPGTSTSATLTGLTR	**112** *	**100.56** *	**103.21** *	**64.94** *	**117.15** *	**128.81** *
Gelsolin, isoform 1	TGAQELLR	−16.51	−25.68	**−21.24** *	−21.98	−21.58	−18.16
Haptoglobin beta chain	VGYVSGWGR	−4.79	−10.49	**−13.00** *	−7.04	−8.07	−9.94
Hemopexin	NFPSPVDAAFR	−9.48	**−15.83** *	**−16.44** *	−13.54	−13.83	**−14.65** *
Heparin cofactor II	TLEAQLTPR	−10.41	**−17.59** *	**−19.00** *	**−**8.69	**−**17.47	**−**14.24
Histidine-rich glycoprotein	DGYLFQLLR	**−**10.27	**−11.50** *	**−14.33** *	**−17.66** *	**−**10.05	**−**8.37
Inter-alpha-trypsin inhibitor HC	AAISGENAGLVR	**−**2.22	**−**4.77	**−**7.60	**−**12.13	1.47	**−**5.41
Kininogen-1	TVGSDTFYSFK	**−11.63** *	**−16.86** *	**−17.27** *	**−16.36** *	**−**14.53	**−**14.88
L-selectin	AEIEYLEK	4.26	**−**13.83	**−**4.46	**−**5.22	**−**16.92	**−**5.92
Plasma retinol-binding protein	YWGVASFLQK	**−**1.64	**−**15.30	**−8.95** *	**−**6.48	**−**12.49	**−**11.77
Plasminogen	LFLEPTR	**13.73** *	7.38	**10.00** *	3.16	6.53	**5.33** *
Prothrombin	ETAASLLQAGYK	**21.05** *	10.40	11.47	6.72	10.88	3.19
Serum amyloid P-component	VGEYSLYIGR	**34.79** *	32.64	21.17	12.82	19.36	16.53
Transferrin	EGYYGYTGAFR	**−**9.64	**−15.46** *	**−18.81** *	**−13.68** *	56.69	**−15.27** *
	HSTIFENLANK	**−**8.39	**−14.76** *	**−19.98** *	**−**13.84	**−**49.93	**−18.90** *
Transthyretin	AADDTWEPFASGK	0.93	**−**8.56	**−9.55** *	**−**13.97	**−**9.51	**−**12.59
Vitamin D-binding protein	THLPEVFLSK	**−**9.26	**−24.76**	**−**14.43	**−16.03** *	**−16.99** *	**−**17.91
Vitronectin	FEDGVLDPDYPR	**−**6.50	**−**14.71	**−17.62** *	**−**10.55	**−**9.41	**−15.95** *
Zinc-alpha-2-glycoprotein	EIPAWVPFDPAAQITK	**−17.37** *	**−**7.93	**−17.85** *	**−15.10** *	**−**9.32	**−**13.50

* p<0.05.

** p<0.001.

The lower levels of many of these peptides in plasma from CTAD tubes suggest that platelet activation may be involved in the release of several of these mid- to high- abundance proteins during delayed sample processing, consistent with previous studies reporting the presence of many of the analysed proteins within platelets [14]. Very few of the peptides measured showed higher levels in samples collected in CTAD tubes. The peptide encoding for fibronectin was the only one with consistently higher levels in CTAD samples at all time points, and with the highest percent increase, ranging from 64–128% (Table 3).

The time course analysis of the MRM data revealed that the proteotypic peptides were relatively stable over time when collected in CTAD or k_2_EDTA tubes, since there was no significant change in the levels of peptides between any of the time points analysed. On the other hand, for samples collected in P100 tubes, significantly lower (p<0.05) levels were observed for a small number of peptides as a function of time: the levels of 7 peptides decreased from time 0 to the 2-hour time point and the levels of 5 peptides decreased from time 0 to the 6-hour time point.

#### The levels of cytokines vary with different collection and processing protocols used

**Figure 1:**
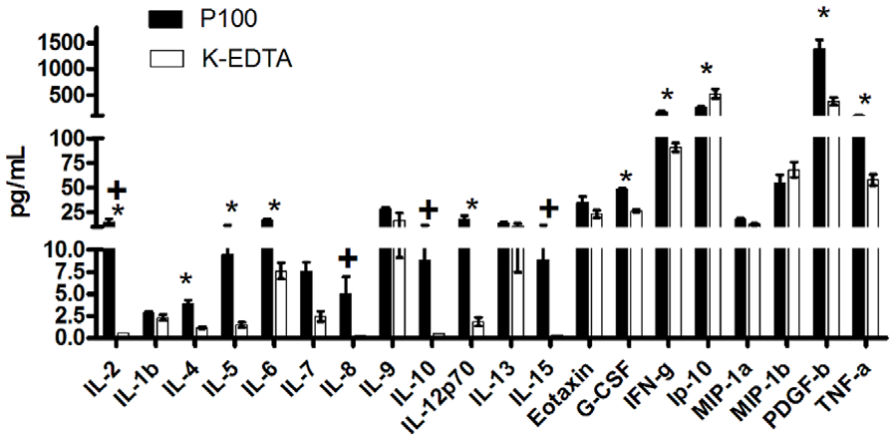
Cytokine levels at time 0. Concentration of cytokines in pg/ml from plasma collected in K_2_EDTA (white bars) and P100 (black bars) tubes processed immediately after collection (T0) using protocol A. The levels of 20 cytokines detected using the Bio-Plex® Assay are shown (RANTES levels not included in the figure, concentration is off scale). Paired t-test comparisons were performed for each cytokine between tube types. * p<0.05, **+** cytokines not detected in K_2_EDTA samples.

Our cytokine analysis was performed using plasma from blood collected in k_2_EDTA tubes and tubes containing k_2_EDTA and protease inhibitors (BD™ P100 Blood Collection Tubes). Each type of tube was processed at several time points after collection (T0, T2, and T6) using the processing protocol as shown in Table 1 (Protocol A or Protocol C). We observed that for at least 80% of the cytokines detected at time 0, the levels were higher in samples collected in k_2_EDTA tubes containing protease inhibitors (P100 tubes) and processed with the initial manufacturer guidelines (protocol A) than in samples collected in k_2_EDTA tubes processed with protocol C, a standard protocol for processing plasma samples (Figure 1). This observation was consistent for all time points analysed (Table S3). Paired t-tests comparing the levels of cytokines measured in both tube types at each of the time points showed significantly (*p*<0.05) higher mean concentrations in P100 plasma relative to k_2_EDTA plasma for 9 cytokines in samples analysed at T0 (mean percent change of 533% (82%–2480%)), for 10 cytokines in T2 samples (mean percent change of 134% (29%–324%)) and for 9 cytokines in T6 samples (mean percent change of 151% (35%–765%)). Interleukins 2, 8, 10 and 15 were not detected in k_2_EDTA samples in at least one of the time points analysed but were detectable at all time points in samples from P100 tubes (Figure 1 and Table S3). Only for IP-10 were higher levels detected in k_2_EDTA samples when compared to P100 at all time points.

Cytokine levels were relatively stable when sample processing was delayed for up to 2 hours; none of the cytokines from k_2_EDTA tubes showed statistically significant changes, while only two cytokines (IL-2 and IL-13) decreased in P100 tubes (Table S4). However, after 6 hours, the mean levels of six cytokines were significantly (*p*<0.05) higher (from 13%–230%) in k_2_EDTA samples relative to time 0, while the concentration of 8 cytokines decreased as a function of time in P100 samples (Figure S1). The magnitude of the change in these cytokines in P100 tubes was less than that observed in k_2_EDTA tubes, with percent changes ranging between 8.5–43% (Supplementary Table S4). Other cytokines did not show statistically significant changes in levels between the time points analysed. In samples collected in k_2_EDTA tubes, interleukins 2, 8, 10, and 15 were below the limit of detection at time 0 but consistently increased with time on the bench and reached detectable levels at the 2-hour or 6-hour time point. None of these changes, however, reached statistical significance. The results of this first assay thus demonstrate that the levels of cytokines measured largely depend on the type of collection tube and the processing protocol used, and can change when processing is delayed after blood collection.

#### Variations in the protocols to process P100 tubes significantly impact the levels of numerous cytokines when processed immediately (time = 0)

**Figure 2:**
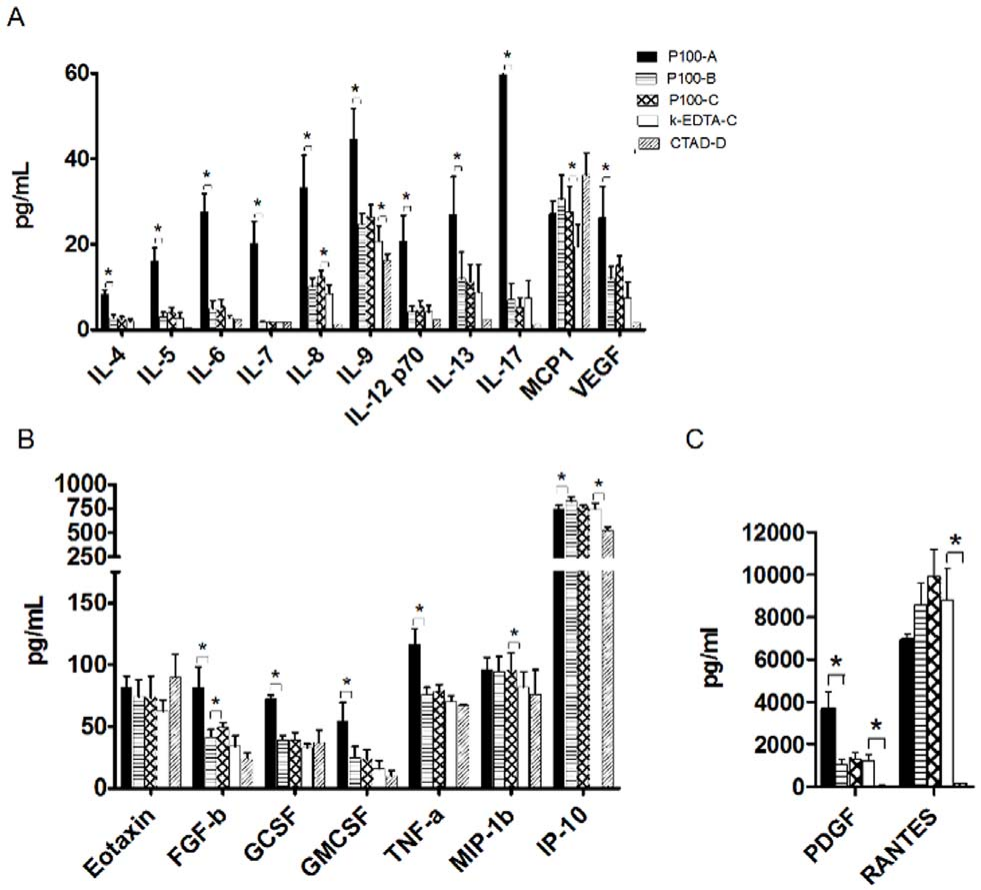
Comparison of cytokine levels between tube types and processing protocols. Concentration of 20 cytokines in pg/ml from plasma collected in K_2_EDTA tubes, P100 tubes processed with different protocols and CTAD tubes. All tubes were processed immediately after collection (T0). Brackets depict groups compared using paired t-tests and for which statistical significance * p<0.05 was found.

The higher levels of cytokines observed in tubes containing protease inhibitors may be related to the fact that different protocols were used to process each type of tube. Therefore, in order to further evaluate P100 tubes, we analysed these tubes again, this time comparing different processing protocols recommended by the company (Protocol A and B), in addition to the protocol used regularly in our laboratory for plasma processing (Protocol C) (Table 1). All samples were processed immediately after blood collection (T0). As mentioned above, 20 cytokines out of the 27 could be measured by the assay. Of the three different protocols used to process P100 tubes, the most significant impact on cytokine levels was observed when we compared the BD initial protocol (protocol A) to the other 2 protocols, *i.e.*, BD updated protocol (protocol B) and protocol C (in-house protocol). As shown in Figure 2, the mean levels of 16 cytokines were higher when samples were processed with protocol A compared to protocol B. In other words, centrifugation under refrigerated conditions for a shorter period of time resulted in statistically significant higher levels for 15 of these analytes (nine of which were interleukins), with percent differences ranging between 50% and 970% (Figure 2A). Protocols B and C are quite similar as the differences between them were mainly differences in centrifugation speed and the addition of a second centrifugation step, without changing centrifugation temperature. These differences did not significantly affect plasma cytokine levels since we found comparable levels for all analytes measured with both methods. No statistically significant differences were observed, except for FGF-b, the levels of which were slightly higher (15%) in samples processed with Protocol C (*p* = 0.03) (Figure 2B). Thus it appears that results with the P100 tubes are largely dependent on the temperature at which centrifugation of the plasma samples is performed. Interestingly, we identified three proteins whose levels were very stable irrespective of the P100 tube processing protocol: these include Eotaxin, MCP-1 and MIP-1b (Figure 2).

#### The presence of protease inhibitors does not significantly impact the levels of cytokines

To directly assess whether the presence of protease inhibitors in the collection tube had an impact on the levels of cytokines, we compared samples collected in k_2_EDTA and P100 tubes processed with identical protocols, in this case, the in-house processing protocol, which avoids cold centrifugation (Protocol C). We found that the addition of protease inhibitors to the blood collection tube only had a modest but significant effect (*p*<0.05) on the mean levels of four of the 20 detected cytokines. The levels of IL-8, IL-9, MCP-1 and MIP-1b were 32%, 21% 29% and 13% higher, respectively, in P100 tubes compared to k_2_EDTA tubes (Figure 2). These differences are unlikely to be clinically significant. These findings demonstrate that the addition of protease inhibitors to plasma collection tubes does not significantly affect the levels of the majority of plasma cytokines when processing is performed at room temperature.

#### Levels for most cytokines are low or undetectable in samples collected in CTAD tubes

In order to directly address the role of platelet activation during processing, we also collected plasma samples in CTAD tubes, which contain additives to prevent platelet activation and degranulation [9], [15]. In our analysis, we found that when samples were collected in CTAD tubes, the levels of IL-4, IL-5, IL-6, IL-7, IL-8, IL-12p70, IL-13, IL-17 and VEGF were below the limit of detection of the present assay in all samples analysed (Figure 2A). Of the 11 cytokines that were measureable in CTAD samples, eight presented lower levels than with any other tube type or protocol used (Figure 2). RANTES and PDGF-b are the two cytokines for which the largest differences in the levels measured were observed. The levels of PDGF-b were 87.3 fold higher in P100 (protocol A) samples (*p* = 0.005) and 29.3 fold higher in K_2_EDTA (Protocol C) samples (*p* = 0.01) when compared to the levels measured in samples collected in CTAD tubes. For RANTES, the range of fold changes was 46.4 fold higher in P100 (Protocol A) samples (*p* = 0.001) and 59.2 fold higher in K_2_EDTA (protocol C) samples (*p* = 0.001) compared to CTAD (Figure 2C). These findings suggest that many of the measured cytokines may become detectable in the plasma because of platelet activation. Interestingly, all of the cytokines that were not detectable in the CTAD tubes were also significantly increased in the P100 tubes when processed using Protocol A compared with Protocol B and C, suggesting that the high levels of these cytokines may be in part due to their release during platelet activation resulting from cold centrifugation in protocol A.

## Discussion

One of the roadblocks in biomarker research is the lack of proper biospecimen quality assessment and quality control. As stated by Rodriguez et al, “The first step in biomarker research that needs to be considered is the development and improvement of biospecimen science [16]. Careful studies on the stability of proteins in biological matrices using different methods of collection, processing, storage and distribution need to be carried out”. The impact of several pre-analytical factors on the measurement of blood cytokines has been previously reported [17]. However it is the first time to our knowledge that a thorough study is performed using both a large multiplex panel (27-plex) and the novel 55-peptide MRM-MS panel to assess the role of factors such as novel blood collection tubes, different processing protocols and delays to centrifugation. Although enzymatic degradation of proteins has been shown to take place during blood collection and processing [18], [19], there has been no consensus as to the recommendations for adding protease inhibitors to blood collection tubes when performing protein biomarker studies [7], [8]. The BD™ P100 Blood Collection Tubes, containing a proprietary cocktail of protease inhibitors, have been reported to inhibit the intrinsic proteolytic degradation of plasma proteins and thus stabilize the blood proteome during collection and processing [20]. Although these tubes may serve to minimize exogenous peptide generation, our present results and those of others [21], [22] have shown that the use of these specialized tubes do not provide enough stability to the plasma proteome to have a significant impact on the levels of peptides measured with different platforms.

We measured 55 mid- to high-abundance proteins with MRM-MS, a mass spectrometry based approach measuring peptides, and 27 cytokines with an antibody-based assay allowing the detection of the whole proteins. In both instances, the levels of the majority of factors measured in P100 tubes were comparable to the levels measured in K_2_EDTA samples when the same processing protocol was used, and these levels remained comparable even when samples were left unprocessed on the bench for up to 6 hours. To our knowledge, this is the first report analysing the effect of protease inhibitors on a large panel of mid/high abundance proteins by MRM-MS and of cytokines. The results from our MRM study showed no significant difference in peptide levels when comparing K_2_EDTA tubes with or without protease inhibitors. These results are in contrast with the report from Randall et al, where five abundant proteins, measured using MRM, showed significant differences when using protease inhibitors [21]. One may argue that the actual protective effect of protease inhibitors on the intact protein is difficult to infer from the measurement of tryptic peptides with MRM-MS. However, tryptic peptides can be further degraded by endogenous proteases, and this degradation could potentially affect the results from MRM studies [23]. The highly comparable levels of peptides from samples collected in P100 and K_2_EDTA tubes in our MRM study suggest that such degradation may not be noticeable at the time points analysed and that protease inhibitors do not confer a significant advantage over regular K_2_2EDTA tubes for MRM-MS studies performed on samples left unprocessed for up to six hours. Thus, in accordance with our conclusions, the Randall study states that the use of specialized collection tubes containing protease inhibitors is not required for MRM assays if strict blood processing protocols are implemented. With the significantly higher cost of P100 tubes (∼$20) vs K_2_EDTA (∼$1), the adoption of this blood collection method becomes less than attractive for clinical biomarker studies, which often require the collection of a large number of blood biospecimens.

Our comparison of protocols to process P100 tubes highlights the importance of standardizing centrifugation conditions. The significantly higher levels of a large number of cytokines measured in samples processed with protocol A is likely to reflect the effect of having a larger number of platelets present in plasma, due to the short centrifugation, and to the release of cytokines from platelets, due to their activation by the cold temperature. Platelets are extremely reactive cells that respond to various stimuli to secrete active compounds, such as cytokines, stored in specific organelles. Shear stress and also exposure to cold temperature during centrifugation alter the morphology of the platelet cells and can result in the activation of a cascade of events eventually resulting in the platelet release reaction [24], [25]. This can significantly impact the results from biomarker studies as seen with the differences in peptide profiles in plasma reported to be caused by the presence and activation of platelets by exposure to low temperatures [7]. Therefore, when samples cannot be processed immediately, it is preferable to keep them at room temperature and avoid refrigeration or keeping them on ice prior to and during processing in order to minimize pre-analytical variability. This is especially critical if cytokines are the biomarkers of interest, since the levels measured will more likely be the result of biospecimen handling rather than of the disease state. Interestingly, MCP1, eotaxin and MIP-1b were found to be very stable and comparable concentrations were detected in samples centrifuged in the cold and at room temperature. Therefore, the levels of these cytokines do not appear to be regulated by platelet activation as further corroborated by their high levels detected in CTAD plasma. Nonetheless, it is important to keep in mind that despite the fact that some proteins may be quite stable under different processing conditions, the majority of cytokines is not.

One of the methods suggested to control for this *ex-vivo* release of cytokines from platelet stores is the collection of blood samples in specialized tubes to inhibit platelet activation. In fact, we found that with the use of citrate-theophylline-adenosine-dipyrimadole (CTAD) tubes the levels of the majority of cytokines were very low or below the limit of detection of the Bio-Plex® assay. These results probably reflect the inhibition of *in vitro* release of growth factors from platelets by CTAD through an increase in cAMP and blockage of calcium-mediated platelet activation as reported by Zimmermann et al [26]. It is important to note that blood samples were collected from healthy volunteers who likely have very low normal levels for most of these cytokines. However in a disease state, a large number of cytokines may reach detectable concentrations. When measuring clinically relevant low-abundance biomarkers known to be stored in platelets, for example VEGF, the prevention of platelet activation by the use of CTAD tubes should help minimize the variability that can arise during blood collection and processing [27], [28]. Our results show that this is not only relevant to the measurement of low-abundance biomarkers such as cytokines, but also to levels of mid- to high-abundance proteins. In fact, platelets also contribute to the circulating levels of many of these abundant plasma factors since many of these are stored within platelet alpha granules [14], [29]. Although lower levels of peptides were detected for many of these proteins in CTAD samples when compared to K_2_EDTA samples, the magnitude of the difference (8%–48% decrease) was not as drastic as the difference observed when comparing cytokines levels between CTAD and K_2_EDTA samples (4%–99% decrease).

The centrifugation of collected blood specimens is likely to be delayed in a clinical setting, due to lack of both human and/or technical resources. Our analysis of samples left un-processed on the bench at room temperature for up to 6 hours showed that there is no impact of such delays in samples that are going to be analysed for mid- to high-abundance plasma proteins using MRM-MS technology. However, we found that increases in the levels of cytokines can be expected due to the release of such factors from platelets, which were activated during centrifugation.

To obtain meaningful results from proteomic blood biomarker studies, it is imperative to standardize all the methods for blood collection and processing. Our recommendations follow those of HUPO and EDRN [7], [8] for the use of k_2_2EDTA plasma for proteomic biomarkers studies, and we find the use of tubes containing protease inhibitors to add extra cost to biomarker studies with little benefit to the protection and stabilization of the blood proteome. As part of any biobanking effort or biomarker study, we strongly recommend the collection of additional blood samples in CTAD tubes to be used for cytokine biomarker studies or other biomarkers likely to be present and released from platelets. Finally, we recommend processing samples immediately after collection and, unlike the EDRN protocol, to avoid refrigeration or centrifugation at cold temperatures to prevent platelet activation. As shown by de Jager et al [17] It is important to also consider the effect of freeze-thaw cycles and long term storage. The collection of datasheets with key information, such as deviations to SOPs, hemolysis, aliquot freeze-and-thaw cycles and length of storage is necessary to ensure the tracking of sample quality and will serve as future reference in the eventuality that results from blood biomarker studies need to be further verified.

## Supporting Information

Figure S1 **Changes in cytokine levels across time points.** Fold change in cytokine levels measured in samples left on the bench up to six hours before processing. Only cytokines showing significant differences (p<0.05) in levels between time points (T0, T2 and T6) in K_2_EDTA tubes (panel a) and P100 tubes (panel b) are depicted.
(TIFF)

Table S1 **List of SIS peptides and technical variability.**
(XLS)

Table S2 **MRM acquisition parameters.**
(XLSX)

Table S3 **Cytokine levels in P100 and K_2_EDTA tubes across time points.**
(XLS)

Table S4 **Change in cytokine levels between time points.**
(XLS)
