# Supplementary figures and images for: The Effect of Pre-Analytical Variability on the Measurement of MRM-MS-Based Mid- to High-Abundance Plasma Protein Biomarkers and a Panel of Cytokines
Source: PLoS One. 2012 Jun 6;7(6):e38290. doi: 10.1371/journal.pone.0038290 (PMC3368926; doi:10.1371/journal.pone.0038290)

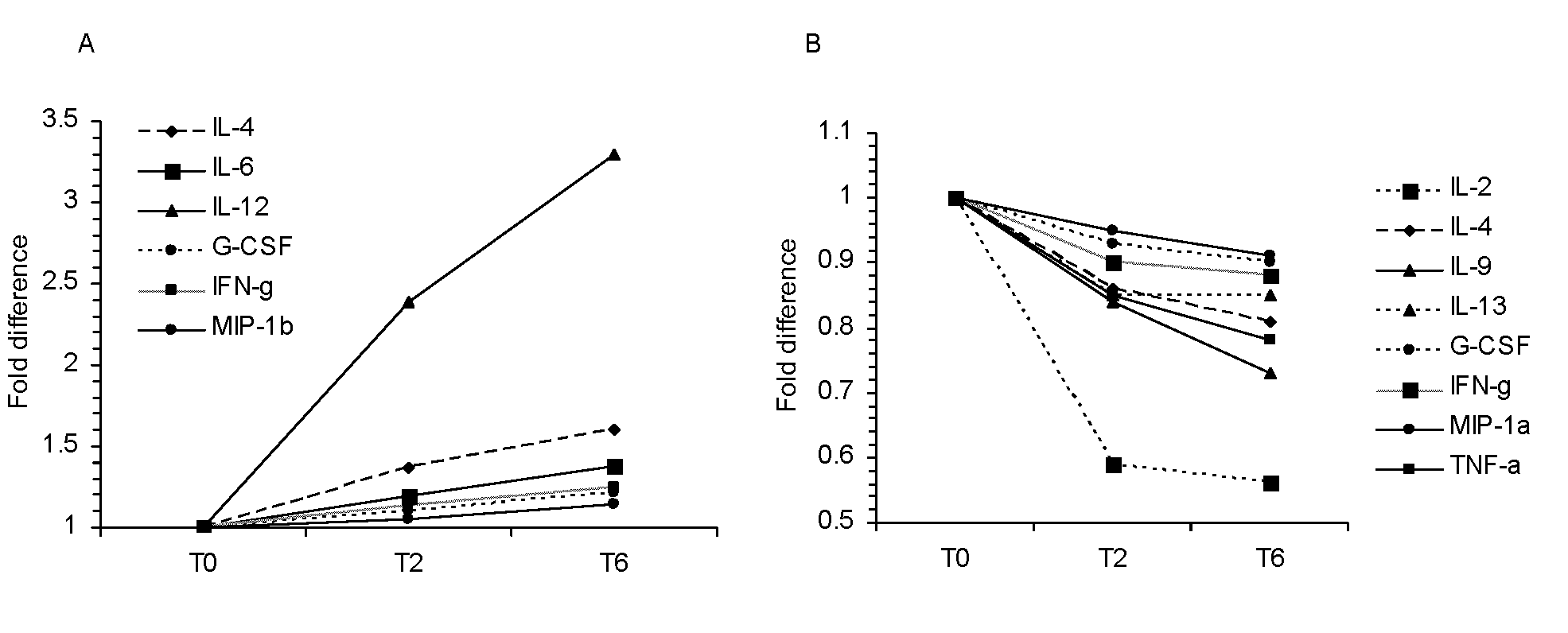

Supplement: Figure S1 — Changes in cytokine levels across time points. Fold change in cytokine levels measured in samples left on the bench up to six hours before processing. Only cytokines showing significant differences (p<0.05) in levels between time points (T0, T2 and T6) in K2EDTA tubes (panel a) and P100 tubes (panel b) are depicted. (TIFF) [file pone.0038290.s001.tif]
